# Chemical Composition and Thermogravimetric Behaviors of Glanded and Glandless Cottonseed Kernels

**DOI:** 10.3390/molecules27010316

**Published:** 2022-01-05

**Authors:** Zhongqi He, Sunghyun Nam, Hailin Zhang, Ocen Modesto Olanya

**Affiliations:** 1 United States Department of Agriculture-Agricultural Research Service, Southern Regional Research Center, 1100 Robert E Lee Blvd., New Orleans, LA 70124, USA; sunghyun.nam@usda.gov; 2 Department of Plant and Soil Sciences, Oklahoma State University, Stillwater, OK 74078, USA; hailin.zhang@okstate.edu; 3 United States Department of Agriculture-Agricultural Research Service, Eastern Regional Research Center, Wyndmoor, PA 19038, USA; modesto.olanya@usda.gov

**Keywords:** cottonseed, fourier transform infrared spectroscopy, glandless, scanning electron microcopy, thermogravimetric analysis

## Abstract

Common “glanded” (Gd) cottonseeds contain the toxic compound gossypol that restricts human consumption of the derived products. The “glandless” (Gl) cottonseeds of a new cotton variety, in contrast, show a trace gossypol content, indicating the great potential of cottonseed for agro-food applications. This work comparatively evaluated the chemical composition and thermogravimetric behaviors of the two types of cottonseed kernels. In contrast to the high gossypol content (3.75 g kg^−1^) observed in Gd kernels, the gossypol level detected in Gl kernels was only 0.06 g kg^−1^, meeting the FDA’s criteria as human food. While the gossypol gland dots in Gd kernels were visually observed, scanning electron microcopy was not able to distinguish the microstructural difference between ground Gd and Gl samples. Chemical analysis and Fourier transform infrared (FTIR) spectroscopy showed that Gl kernels and Gd kernels had similar chemical components and mineral contents, but the former was slightly higher in protein, starch, and phosphorus contents. Thermogravimetric (TG) processes of both kernels and their residues after hexane and ethanol extraction were based on three stages of drying, de-volatilization, and char formation. TG-FTIR analysis revealed apparent spectral differences between Gd and Gl samples, as well as between raw and extracted cottonseed kernel samples, indicating that some components in Gd kernels were more susceptible to thermal decomposition than Gl kernels. The TG and TG-FTIR observations suggested that the Gl kernels could be heat treated (e.g., frying and roasting) at an optimal temperature of 140–150 °C for food applications. On the other hand, optimal pyrolysis temperatures would be much higher (350–500 °C) for Gd cottonseed and its defatted residues for non-food bio-oil and biochar production. The findings from this research enhance the potential utilization of Gd and Gl cottonseed kernels for food applications.

## 1. Introduction

**Figure 1:**
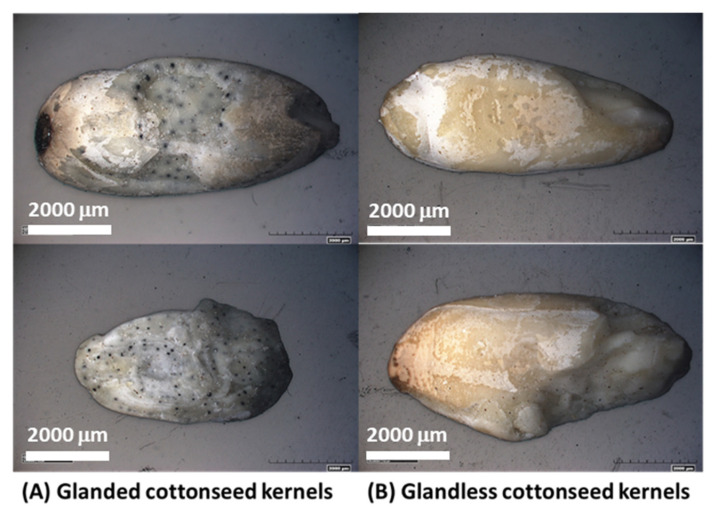
Images of glanded (**A**) and glandless (**B**) cottonseed kernels. The small black dots appearing on the kernel surfaces of sample (**A**) are the gossypol glands.

Cottonseed is a major product of cotton (*Gossypium* spp.) crops after fiber harvest [1,2]. The current global cottonseed production is estimated at 42 × 10^6^ Mg annually [3]. Currently, cotton fiber accounts for 85–90% of the value of the crop while cottonseed accounts for the rest, a small share even though there are 150 kg of cottonseed produced for every 100 kg fiber ginned [4]. Cottonseeds can be utilized directly or further processed into five products, i.e., linter (8%), hulls (27%), crude oil (16%), meal (protein and carbohydrate, 45%), and waste (4%) [4,5]. Refined cottonseed oil is edible, and has been used in the food industry as a cooking oil or an ingredient in salad dressing, shortening, and mayonnaise, so it contributes currently to the major income of cottonseed processing [4,6]. However, the nutrient-rich whole cottonseed and its non-oil products cannot be used directly for human and other monogastric animal consumption, due to the presence of the toxic compound gossypol [C_30_H_30_O_8_, 1.l,6,6,7,7-hexahydroxy-5,5-diisopropyl-3,3-dimethyl-(2,2-binaphthalene)-8,8-dicarbaldehyde, or 2,2′-bis-(formyl-1,6,7-trihydroxy-5-isopropyl-3-methylnaphthalene] stored mainly in the pigment glands [7,8]. Gossypol not only can lead to liver damage and even fibrosis itself, but also can combine easily with the ε-amino group of lysine of protein fractions, resulting in a decrease of lysine activity, which makes lysine become the first limiting amino acid in nutrition [9,10]. Thus, research efforts have been made to produce a new type of cottonseed in which gossypol is present only in trace amounts so that the cottonseed and its value added products can be used for animal feeds and human foods [11,12,13]. To distinguish the two types of cottonseeds, the new type is called “glandless” (Gl) while the traditional type is called “glanded” (Gd) cottonseed (Figure 1). While there is plenty of research on characterization and utilization of Gd products [2,14,15], research on Gl samples is quite limited. Generally, Gl protein-based adhesives have shown similar bonding performance as their Gd counterparts [16]. Both Gd and Gl kernels contained some bioactive ingredients and antioxidant activities [17,18,19]. However, peptide profiling indicated that there might be some differences in the distribution patterns of the storage proteins in Gd and Gl preparations, although more quantitative studies are needed [9,20]. Compared to soy protein, Gl protein isolate showed lower water-holding and oil-holding capacity but had similar gelation properties, elevated foaming capacity at high pH values, and greater emulsion stability [21]. With a trace content of toxic gossypol, Gl cottonseed is especially promising if processed for food applications, such as roasted nut-like products and cottonseed butter although no viable processing details have been reported [22,23]. Reyes-Jáquez et al. [24,25] formulated and evaluated Gl-meal/corn flour snacks by extrusion cooking. Shrimp feed with Gl cottonseed meal as a protein source was made by extrusion and its structural, rheological and calorimetric properties evaluated, showing the Gl meal-based shrimp feed is a reasonable option to decrease feeding costs [26,27]. Specifically, Gl cottonseed protein could replace up to 75% fishmeal protein in the diet of southern flounder without compromising its growth potential [28]. In other words, Gl cottonseed could be an inexpensive protein source for the commercial culture of southern flounder and other finfish species.

Fourier transform infrared (FTIR) spectroscopy is one of the non-destructive instrumental techniques widely used in applied cotton fiber, cottonseed, and other cotton biomass research [29,30,31,32]. Cottonseed-derived oil [33,34], protein [35,36] and carbohydrate products [31,37] have shown different FTIR features. Specifically, FTIR intensities of 1665 to 1680 cm^−1^, 1646 to 1660 cm^−1^, 1638 to 1645 cm^−1^, and 1615 to 1637 cm^−1^ have been used to evaluate cottonseed protein’s secondary structures (β-turns, α-helices, random coils, and β-sheets) [38,39,40]. Sun et al. [41] used FTIR to detect the chemical and conformational changes between transgenic cottonseeds and their non-transgenic counterparts. While not much difference was observed in original FTIR spectra between these samples, spectral treatments by Fourier self-deconvolution (FSD) and peak-smoothing in the regions between 2000 and 1000 cm^−1^ showed that the differences in band patterns observed between transgenic cottonseeds and their counterparts depended on the varieties used.

A thermogravimetric analyzer (TG) is another advanced instrument used to analyze the mass changes of a sample with increasing temperatures and time [42]. Heat treatments such as roasting and frying are frequently employed in the food processing of seeds and nuts to improve their sensory quality, digestibility, and microbiological safety [43,44,45]. Thus, TG analysis has been applied to detect the mass loss of various volatile compounds as well as appropriate roasting temperatures for heat treatments [44,46,47]. The coupling of TG with FTIR (i.e., TG-FTIR) has provided a unique capability to obtain the transient mass loss and evaluation of the volatile functional groups of a sample, which could not be obtained by the TG and FTIR individually [42,48]. In previous research, this technology revealed the presence of phenols, esters, alcohols, and aldehydes as the volatile compounds in hazel sterculia (*Sterculia foetida* L.) seeds [49]. Examination of defatted safflower seeds by TG-FTIR analysis found that CO_2_, C_6_H_5_OH, and C=C-containing compounds were the main pyrolysis gas products of defatted safflower seeds [50]. TG-FTIR observations have shown that the devolatilization sensitivity to heating rate followed the order of hemicellulose > starch > oil > cellulose ≈ protein > lignin [51]. In a characterization of five herb essential oils, the TG of the essential oils and the TG-FTIR analyses of the evolved gases showed that the essential oils’ thermal behavior was due to the volatilization of the major components in these herbs without thermal degradation [52].

Similarly, data on the thermal behavior and stability will be useful for safe and proper handling of cottonseed during product formulations. To the best of our knowledge, there are only limited TG-FTIR studies of cottonseed with the purpose of pyrolysis applications so far [53,54,55]. Thus, in this research work, chemical analysis, FTIR, and TG-FTIR were applied to evaluate the thermochemical properties of Gd and Gl cottonseed kernels. Knowledge and data obtained from the thermal behavior and stability are useful for a safe and proper handling of cottonseed during product formulation for food and non-food applications. Therefore, the primary objective of this research was to determine the thermal properties and stability of Gd and Gl using TG-FTIR analysis.

## 2. Results and Discussion

### 2.1. Chemical Composition of Gd and Gl Kernels

Selected chemical components of Gd and Gl cottonseed kernels are listed in Table 1. These data are generally in the range described in the literature [56,57,58,59]. The unique features in the presence or absence of gossypol are clearly demonstrated by the difference in gossypol content between the two types of cottonseeds. These data indicated that Gl kernels, but not the Gd kernels, meet FDA’s criteria as human food products with free gossypol < 450 ppm [2,23]. Otherwise, the chemical components of Gd and Gl were quite similar. In both types of kernels, oil and protein accounted for about 40% of the total biomass. The carbohydrates, moisture, and mineral ash constituted the remaining biomass. Even though a significant (*p* < 0.05) difference was observed, the starch content was low in both Gd and Gl samples, consistent with the data in the literature [22,60]. While cottonseed could be a source of human food and animal feed for low-starch diets [22], external addition of starch may promote the processing and dietary properties of cottonseed products [24,26]. Statistically significant (*p* ≤ 0.05) differences were also observed in the contents of protein and total phosphorus between Gd and Gl kernels. The similar significant impact seems reasonable as phosphorus in cottonseeds is generally present in the phytate form [61] and positively correlated with cottonseed protein content [62]. While the composition of major components of the Gd and Gl cottonseeds are available in the literature [22,23], Table 1 also compared the mineral contents between the two types of cottonseeds. Twelve of the thirteen minerals (except for Al) are essential elements. Aluminum is a non-essential element whose presence is of concern to both human and ecosystem health [56,63]. The general similarity of the mineral levels between the two cottonseeds implied that the decrease of gossypol in Gl cottonseed did not dramatically alter the mineral composition of cottonseeds. Therefore, the quality of mineral nutrients of the cottonseeds was not impacted by the reduction of the toxicity of high gossypol content.

### 2.2. Microstructure of Gd and Gl Cottonseed Kernels

**Figure 2:**
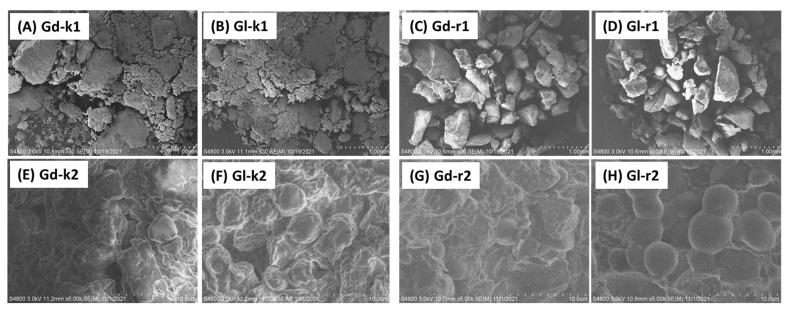
SEM images of Gd and Gl kernels (Gd-k and Gl-k) and their residues (Gd-r and Gl-r) after sequential 100% hexane and 80% ethanol (in water) extractions. Bar distances are 1.00 mm and 10 μm, respectively, for series 1 (upper row panel (**A**–**D**)) and 2 (lower row panel (**E**–**H**)) images.

Figure 2 shows the SEM images of the Gd and Gl kernel particles and their extraction residues at two different scales. The SEM images of both Gd-k and Gl-k appear to have no apparent differences. The images at the larger scale (1 mm) showed that the powder particles of both kernels seemed to form sticky aggregates. The images at the smaller scale (10 μm) revealed that the morphology of these kernel powders had stacked granules of irregular spherical shapes. By careful comparison of the images of Gd-k2 and Gl-k2, it can be seen that the granule surfaces of the latter are smoother than the former. These micrographs indicate that the presence of gossypol did not result in any difference in micromorphology of the two types of cottonseed powders. On the other hand, some differences in the microstructures were observed in the kernel residues after hexane and 80% ethanol extraction. In a comparison to Gd-k1 and Gl-k1, the images of the Gd-r1 and Gl-r1 appeared to be more separated and with large chunks disrupting the outer layer of the fibers, similar to the microstructures of those defatted cottonseed and soy meal products [64,65]. Thus, we attributed this micromorphological difference to the oil component. Another observation was that almost all granules in the Gl-k2 image were round and smoothy, but there were only a few such round-shaped granules in the microstructural matrix in the Gd-r2 image (Figure 2). More data from future research are needed to confirm if the microstructural difference was due to the effects of a broken gossypol gland wall matrix which is not hexane extractable [66].

### 2.3. ATR FTIR Spectra of Gd and Gl Kernels and Their Extraction Residues

**Figure 3:**
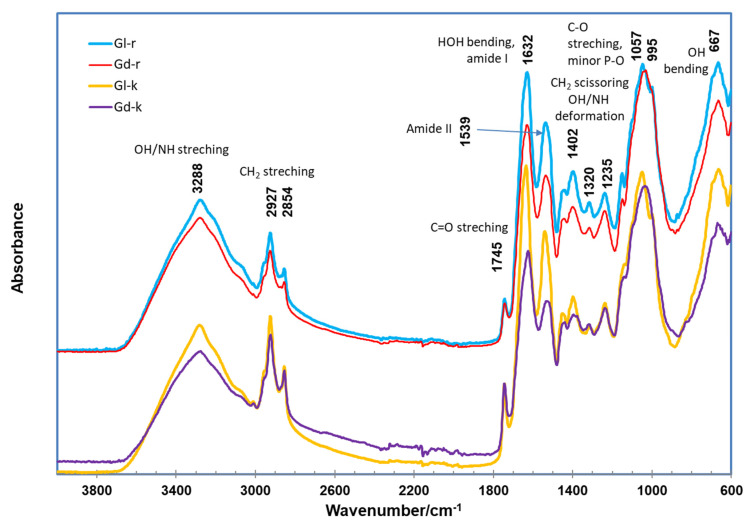
ATR FTIR spectra of Gd and Gl kernels (Gd-k and Gl-k) and their residues after 100% hexane and 80% ethanol (in water) extraction (Gd-r and Gl-r).

The ATR FTIR spectra of the four samples are shown in Figure 3. As per the previous band assignments of cotton biomass sample research [31,32,38,44,67], the typically broad and strong band around 3288 cm^−1^ was due to the O-H or N-H stretching modes of carbohydrates, adsorbed water, and proteins. The twin or triple bands at 2927 and 2854 cm^−1^ were assigned to hydrophobic CH_2_ asymmetrical and symmetrical stretching vibrations from the oil portion in cottonseed. The band at 1745 cm^−1^ was contributed by the C=O stretching modes of carbonyl groups. The bands at 1632, 1539, and 1235 cm^−1^ were attributed to amide I (C=O stretching), II (CN stretching, NH bending), and III (CN stretching, NH bending) bands of proteins, respectively. The bands at 1070–995 cm^−1^ were assigned mainly to carbohydrates with a possible minor contribution of phosphate in cottonseeds. Similar to the chemical composition, the spectral features of Gd and Gl samples were similar with some differences only in band strength. Therefore, gossypol did not make any remarkable visual differences in the FTIR spectral features of Gd and Gl samples. This is only due to the low gossypol content (0.375%), and also due to the fact that the FTIR spectrum of gossypol possesses no unique spectral features, rather than the general band characteristics of aromatic rings, aldehydes, and phenolic hydroxyl groups [68]. A complicated mathematical model is needed to reveal the gossypol-related difference between Gd and Gl samples, such as a partial squares method based on all the band data from 3700–2400 cm^−1^ and from 1900–750 cm^−1^ for determination of gossypol in cottonseed oil samples [68].

**Figure 4:**
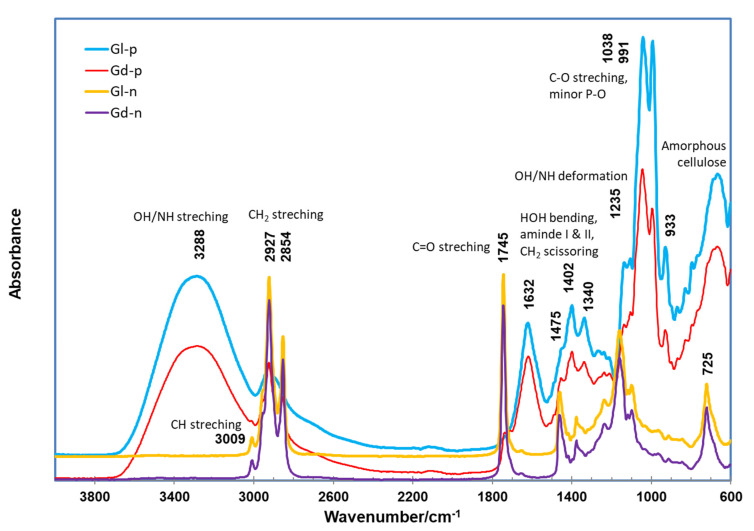
ATR FTIR spectra of the extracts of Gd and Gl kernels by nonpolar 100% hexane (Gd-n and Gl-n) and polar 80% ethanol (Gd-p and Gl-p).

After sequential 100% hexane and 80% ethanol extraction, the spectra of the residues (Gd-r and Gl-r) (Figure 3) of the two kernels showed relatively weaker peaks around the 2927 and 2854 cm^−1^ region, compared to the unextracted kernel samples (Gd-k and Gl-k), apparently due to the removal of the oil components [32,69]. This observation was further confirmed by the ATR FTIR spectra of the hexane extracts (Gd-n and Gl-n) (Figure 4), which are typical for seed oil samples with strong bands at 2927 and 2854 cm^−1^ regions as well as at 1745 cm^−1^ [32,70]. Compared to the Gd-k and Gl-k samples, Gd-n and Gl-n showed a more apparent minor peak at 3009 cm^−1^. This peak was associated with the stretching vibration of CH cis-olefinic groups [45]. In comparison to their untreated kernel samples, no other apparent changes in FTIR features of the extraction residues (Gd-r and Gl-r) were observed, leading to their features being similar to those of defatted cottonseed meal products [71].

As there were only minor spectral differences between the two cottonseed samples, we further examined the spectral features of the hexane and methanol extracts of Gd and Gl samples to enhance the understanding of the chemical composition of the specific extractable nonpolar and polar ingredients in cottonseeds. The spectra of the ethanol extracts (Gd-p and Gl-p) were totally different from the features of nonpolar extracts Gd-n and Gl-n, and were characterized by the highest bands of carbohydrates at the 1038–991 cm^−1^ region (Figure 4). While no FTIR information on the ethanol extracts of cottonseed kernels is available in the literature, HPLC-MS analysis revealed the ethanol extracts of Gd and Gl cottonseed with 27 peaks and mass values equivalent to possible flavonols or derivatives and 16 peaks possible matched to apiosyl, rhamnosyl, and glucosyl-derivative mass [17]. Ultrahigh resolution mass spectrometry may be needed to identify definitely these compounds in the ethanol extracts of Gd and Gl kernels [72].

### 2.4. Thermogravimetric Observations

**Figure 5:**
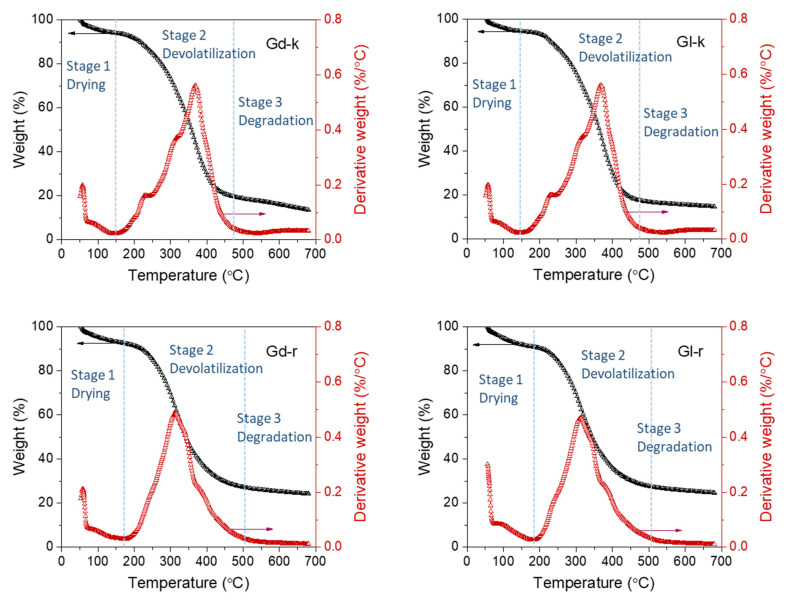
Thermograms of Gd and Gl kernels (Gd-k and Gl-k) and their residues after hexane and 80% ethanol extraction (Gd-r and Gl-r). Left y axis: TG plots. Right y axis: DTG plots.

The TG plots of the Gd and Gl samples are shown in Figure 5, respectively. The general features of these TG and DTG plots were the typical three-stage (drying, de-volatilization, and char formation) weight loss of the testing samples over the heating temperature range from 50–700 °C. Analysis of the first derivative curves of the TG measurement (DTG) of the kernel samples showed more clearly that the second stage of decomposition processed with three substeps had maximum temperature peaks of around 215 (shoulder), 315 (shoulder), and 380 °C (major peak) (Figure 5). This thermal behavior of cottonseed kernels was more complex than the relative sample DTG curves of cottonseed oil [73], protein [36], and fiber [30]. Furthermore, the DTG curves of the extraction residues (Gd-r and Gl-r) showed different decomposition characteristics with the unnoticeable first shoulder (215 °C), a major peak at 380 °C, and switching the peak at 380 °C to a shoulder. Thus, in addition to the attributed common decomposition of hemicellulose, cellulose, and lignin assigned for cottonseed and other natural products [48,53,55], the differences of DTG maximal temperature peak shape between the kernels (Gd-k and Gl-k) and extraction residues (Gd-r and Gl-r) indicated that the oil component should be a major contribution of the decomposition around 380 °C. Those ethanol extractable compounds volatilized or decomposed around 215 °C. Protein components were decomposed in a wide range of temperatures from 215 to 380 °C [36,74].

The thermal gravimetric observations of the Gd and Gl samples were similar, but not exactly like those of cottonseed samples in the literature [53,54,55]. The differences were due to the fact that dehulled kernels were used in the study while whole cottonseeds were used in the three studies reported previously. Similar to the thermal behavior of hazel seeds [49], the first stage of drying of our cottonseed kernels at 90 °C was the loss of moisture. Calculating from the weight loss from 50 °C to 144 °C as the onset of decomposition, the evaporation (drying) of moisture contents of Gd-k was about 5.9% weight loss (Table 2). In the second stage of devolatilization, the rapid decomposition of the sample occurred between 150 and 472 °C representing about 70.4% (i.e., 80.3%(WL_e_) − 5.9%(WL_o_)) weight loss due to devolatilization, breaking of chemical bonds, and destruction of the parent molecular skeletons. In the last stage above 472 °C (degradation), decomposition of char (19.7%) occurred with the formation of secondary pyrolytic vapors, but some char or ash remained (<16.1%). While the Gl-k sample demonstrated similar thermogravimetric properties, the values of four of the seven parameters measured (Table 2) were significantly different (*p* ≤ 0.05) from the corresponding values of the Gd-k sample. Compared to Gd-k, Gl-k kernels started the devolatilization stage at a higher temperature (147 °C), but the ending temperature (469 °C) was lower. Despite this, there was more weight loss of Gl-k (82.2%) than Gd-k (80.3)%. In contrast, the *T_o_* values differed only significantly between the residual Gd-r and Gl-r samples. Therefore, the thermogravimetric properties of the two extraction residues were more similar to each other than the Gd and Gl kernels themselves. This observation implied that the extractable components in Gl kernels were less thermally stable. However, we did not establish a correlation of the thermogravimetric difference with any of these components between Gd and Gl-samples.

High-quality cottonseed and its derived products/byproducts have been developed as animal feeds and human foods [23,26,75,76,77,78]. These low quality or non-edible cottonseed, oil, and other byproducts may be used for raw materials of bio-fuels and biochars [54,55,79,80,81]. These applications are frequently involved with various heat pre-treatments (such as low-temperature roasting/frying and mid- or high-temperature pyrolysis). While there are no clear-cut point of temperature settings for each heat treatment, thermogravic observations would be helpful in understanding the chemicophysical changes of the cottonseed under a given heating temperature range. Specifically, the temperature of the devolatilization stage would be a critical reference for product optimization (such as flavors of roasting and yield of bio-oil/bio-gas) as vaporization of the volatile components of the biomass occur. Thus, we applied TG-FTIR to elucidate more information on transient mass loss and evaporation of the volatile functional groups of the two cottonseed samples during the stage (Section 2.5).

### 2.5. TG-FTIR Observations

**Figure 6:**
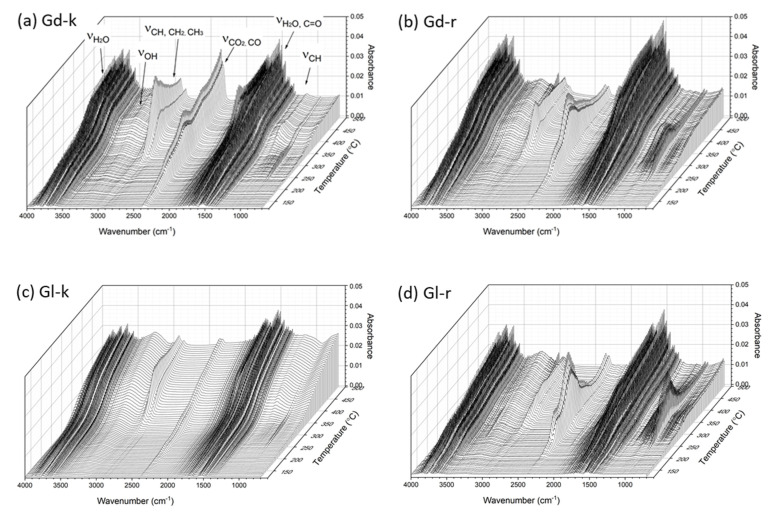
TG-FTIR spectra of Gd and Gl kernels (Gd-k and Gl-k) and their residues after 100% hexane and 80% ethanol extraction (Gd-r and Gl-r).

The three-dimensional TG-FTIR spectra with the coordinates of absorbance, wave number, and temperature of the four samples are shown in Figure 6. This methods of TG analysis coupled with IR spectroscopy detected the real-time gaseous products that evolved during the pyrolysis process [48]. The intensive FTIR signals of the evolved gases were detected within a temperature range of 150 and 500 °C which is consistent with TGA measurements (Figure 5). The presence of H_2_O was demonstrated by O–H stretching vibrations between 4000–3400 cm^–1^ and the sharp band at 1514 cm^–1^ [48]. The broad band in the range of 3000–2700 cm^–1^ represented hydrocarbon gases, mainly CH_4_, and the remarkable peak and a small band between 2400 to 2000 cm^−1^ represented the C = O stretching vibrations of CO_2_ and CO [49,53]. The presence of aldehydes, ketones, and acids was indicated by the band at 1776 cm^–1^. The broad bands between 1460–1365 cm^–1^ and 1200–1000 cm^–1^ were attributed to the presence of C–H alkanes, esters, and alcohols. In addition, the band between 1200 to 1000 cm^–1^ further confirmed the presence of alcohol in these gaseous products [48,49].

The main differences between the TG-FTIR spectra of the Gd-k and Gl samples were the presence of much stronger peaks in three regions of 3000–2700 cm^–1^ (CH_4_), 2400–2300 cm^−1^ (CO_2_, CO) and the minor bands around 1000 cm^−1^ (alcohol) with the Gd-k sample. This observation implied that the biomass components in Gd kernels decomposed faster than those in Gl kernels. However, the spectra of the residues after hexane and ethanol extractions of the glanded and glandless cottonseed samples were more similar to each other. Indeed, the FTIR bands in the three above regions were somewhat stronger in Gl-r sample than Gd-r samples. As the two-step extractions mainly removed the major oil components and minor flavonols or similar compounds alike [17], it seemed that the oil component made the difference in TG-FTIR spectra of Gd and Gl samples. As free gossypol is lipophilic [68], we hypothesize that it was gossypol dissolved in oil that promoted the faster devolatilization in the glanded cottonseed kernel sample GD-k although the hypothesis should be further examined with more experimental data. The TG-FTIR spectra also showed that the CO_2_ and CO bands (2400–2300 cm^−1^) reached the highest point around 315 ^°^C, but the CH_4_ peak (3000–2700 cm^–1^) was at its highest point later at approximately 375 ^°^C. The latter CH_4_ peak was also weaker in the oil-extracted residual samples (Gd-r and Gl-r) than their corresponding original kernel samples (GD-k and Gl-k), indicating that CH_4_ detected in the TG-FTIR spectra was mainly from the oil component of cottonseed. In contrast to that, these CO_2_ and CO products that appeared at early stages should be mainly from carbohydrates (cellulose, hemicellulose, starch) [48,51]. Compared to the kernel samples, the relatively stronger multiple bands from 1500 to 700 cm^−1^ in Gd-r and Gl-r) indicated easier decomposition of protein components after oil removal [51]. This observation was supported by N-enriched compounds of the defatted cottonseed meal bio-oil which were the condensed gaseous products of pyrolysis [54,80]. In other words, it was difficult to pyrolyze the biomass rich in oil and lignin but easy to pyrolyze the biomass rich in cellulose, starch, hemicellulose, and protein [51].

Similar to other seeds or nuts for food applications [45,47,82,83,84,85], roasting is a necessary heating process for enhanced use of cottonseed kernels as nutrient food products [23]. While roasting temperatures were reported in the range of 100 to 160 °C for various plant-based butters [86], the negligible CO_2_ and CO bands (2400–2300 cm^−1^) in TG-FTIR spectra of Gd-k and Gl-k below 215 °C of the first shoulder in DTG curves (Figure 5) suggested that the cottonseed kernels could be roasted in the temperature range without significant occurrence of decomposition (carbonization). On the other hand, the optimal roasting temperature would be lower than or around 140–150 °C in the first stage of drying or dehydration (Figure 5) to reduce evaporation of some volatile flavor compounds [43]. The TG-FTIR spectra also confirmed that 350 to 500 °C were optimal pyrolysis temperatures of cottonseed kernels and their defatted residues (i. e., defatted meal or cake) for bio-oil and biochar production [55,80,87] with some flexibility of higher temperatures (e.g., 600–700 °C) [53,54].

## 3. Materials and Methods

### 3.1. Cottonseed Source and Treatment

**Figure 7:**
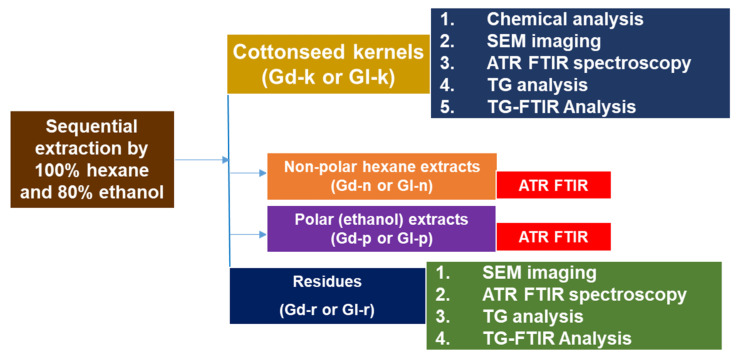
Flow chart of the sample processing, experimentation, and analysis performed in this work. Gd series, glanded cottonseed samples; Gl series, glandless cottonseed samples.

The Gl cottonseed of the NuMex series was provided by Cotton, Inc. (Cary, NC, USA) [9,88]. These seeds were dehulled mechanically by cracking with a 20.32 cm plate mill, and then separated with a vibration shaker. The kernel products were further cleaned by passing the material through a laboratory aspirator to remove the non-kernel material. The presence of contamination of Gd cottonseeds in the Gl products due to cross fertilization by windblown or insect-carried pollen of Gd varieties was noticeable [23]. These Gd kernels were hand-picked or removed and used as the comparative Gd sample in this study. Both Gl and Gd kernels were then ground for 3 min in a stainless-steel jar of Waring Commercial Blender (Model WF2211214, Torrington, CT, USA). The experimental procedure, methodologies, and sample labelling are shown in Figure 7 and described in the sub-sections below.

### 3.2. Non-Polar and Polar Solvent Extraction

Cottonseed kernels were subjected to sequential extraction by 100% reagent grade hexane [89,90] and 80% reagent grade ethanol in water [17,83]. Grounded Gd and Gl kernels (5.00 g each) were placed in 50 mL centrifuge tubes with 15 mL of hexane and extracted overnight (18 h) at room temperature (26 °C) under rotary shaking (60 rpm). Those tubes were then centrifuged for 30 min at 5 °C and 2500× *g*. After centrifugation, the supernatant in each tube was carefully removed and the pellets (residual parts) were extracted one more time following the same procedure mentioned above. The residual parts were subsequently washed twice with hexane (5 mL each). The supernatants and washing solutions of each tube were pooled and placed in a venting hood to evaporate hexane out at room temperature. The non-evaporated part was the nonpolar oil fraction of the cottonseed kernels. The dried residual pellets were further extracted twice by 80% ethanol (15 mL each) following the same procedure as in the hexane extraction. Both the ethanol supernatants (polar extracts) and the extracted residues were dried in a vacuum oven at 40 °C to constant weights.

### 3.3. Microstructural Imaging Analysis

For scanning electron microscopy (SEM), a thin layer of sample particles (grounded cottonseed kernel or the extraction residues) was gently attached to an 8 mm × 12 mm double-side sticky carbon tape on an aluminum stud. The butter sample was coated with 3 nm thickness of carbon using a Cressington 208HR Sputter Coater (Watford, England, UK). The cottonseed kernel samples were observed and imaged with a Hitachi S-4800 Field Emission Scanning Electron Microscope (Hitachi, Japan), operating at 3 kV [30]. Microscopic images were done on multiple samples with different scales and the representative images were reported.

### 3.4. Chemical Analysis

All chemical analysis and data were reported on a dry weight basis. Moisture content was determined as the loss in weight upon drying a sample in a forced draft oven at 105 °C for 5 h [91]. Both (+) and (−)isomers of gossypol were detected by a modified procedure of AOCS Recommended Practice Ba 8a-99, using about 100 mg sample for each analysis [92,93]. Briefly, 100 mg of ground sample was weighed into a 12 mL screw-cap test tube. A complexing reagent (2 mL) consisting of 2/10/88 (*v/v/v*) R-(-)-2-amino-1-propanol, glacial acetic acid, and dimethylforamide was added. The tube was then heated at 95 to 100 °C for 30 min to convert gossypol’s aldehyde groups into Schiff ’s bases with the chiral amine. The formed gossypol complexes were detected on-line at 254 nm after they were separated on a Hewlett-Packard Series 1100 HPLC system equipped with a photodiode array detector (Palo Alto, CA, USA). Total gossypol content was computed as the sum of these isomers. Starch content was measured per Sigma–Aldrich starch assay kit (SA20, Sigma–Aldrich, St. Louis, MO, USA). Crude protein content in the samples was calculated by multiplying the total N by a factor of 6.25 [57]. Oil content was estimated per the yield of hexane extraction [90]. Acid detergent fiber (ADF) and acid detergent lignin (ADL) were determined using the filter bag methods with an Ankom Fiber Analyzer (Ankom Technology, Macedon, NY, USA). The kernel samples were first pre-extracted by acetone before the fiber analysis to remove the inherent oil [94].

The elemental composition of the samples was analyzed following acid digestion. Briefly, 0.50 g of grounded sample was mixed in 10.0 mL of concentrated HNO_3_ for 1 h in a HotBlock™ 200 digestion system (Environmental Express, Charleston, SC, USA). The sample was then heated to 115 °C for 2.25 h. The concentrations of 13 elements (i.e., Al, B, Ca, Cu, Fe, K, Mg, Mn, Na, Ni, P, S, and Zn) in these digests were determined by a Spectro CirOs ICP spectrometer (Mahwah, NJ, USA) [57]. The concentrations of total N in each sample were determined using a LECO Truspec dry combustion Carbon/Nitrogen Analyzer (LECO, St. Joseph, MI, USA). Ash content was determined by measuring the residual mass of a sample (1.0 g) after heating in a muffle furnace at 550 °C for 4 h [95]. Samples were replicated (3×) and chemical analyses were repeated.

### 3.5. Instrumental Analysis

ATR FTIR spectra were measured using a Vertex 70v FTIR spectrometer (Bruker Daltonics, Billerica, MA, USA) equipped with a MIRacle ATR accessory (Pike Technologies, Fitchburg, WI, USA) that incorporated a diamond crystal plate as the reflector. The ground solid samples or oily-like extracts were placed on the ATR crystal surface and secured with a metal clamp to ensure a reproducible pressure which was applied to the samples to achieve intimate contact with the ATR crystal. The spectra were collected over the range of 4000–600 cm^−1^ at 4 cm^−1^ resolution and with 16 scans. All spectra were normalized and presented in absorbance [80].

Thermogravimetric analysis was carried out using a TGA Q500 thermal gravimetric analyzer (TA Instruments, New Castle, DE, USA) under a nitrogen atmosphere. The nitrogen flow into the furnace was maintained at a rate of 90 mL/min. About 8 mg of the sample placed in a platinum pan was heated to 700 °C with a heating rate of 10 °C/min. The evolved gas during the thermal decomposition of the sample was analyzed using TG-FTIR. A TG analyzer was equipped with a gas purge through which pyrolysis vapors were conveyed to a TG-FTIR interface inside the infrared spectrometer. A series of FTIR spectra were collected from 100 °C to 500 °C with 16 scans at a 4 cm^−1^ resolution from replicated samples. Two spectra were obtained every minute [48].

### 3.6. Statistical Analysis

Data are presented in the format of average ± standard deviations. Average values, if significantly different (*p* ≤ 0.05), are indicated by the relevant symbols in Table 1 and Table 2.

## 4. Conclusions

This work showed a comparative investigation of the chemical and thermal properties of glanded and glandless cottonseed kernels. While the whole glanded kernels were full of visible dark gossypol gland dots, scanning electron microcopy was not able to show the microstructural difference between grounded Gd and Gl kernel particles. With only 1.6% of the typical gossypol content of glanded kernels, the tested glandless kernels had slightly, yet statistically significant higher (*p* ≤ 0.05) contents of protein, starch and phosphorus as compared to the glanded sample. Chemical analysis and FTIR spectroscopy showed similar composition in C functional groups and minerals in the two types of cottonseeds. TG analysis showed a typical three-stage process (drying, de-volatilization, and char formation) of weight loss of the tested kernel samples when subjected to temperatures of 50–700 °C. TG-FTIR spectroscopy revealed apparent differences in thermogravimetric properties between the two kernel samples, as well as between the raw and extracted kernel samples as some components in glanded kernels were more prone to thermal decomposition than glandless kernels.

With very low gossypol content, Gl cottonseed and its derived products could be developed as value added animal feeds and human foods, and part of the low quality (or non-edible grade) Gl cottonseed as well as the high-gossypol Gd cottonseed products can be used as feedstock for bio-energy and biochars. These applications all need certain heat treatments (such as low-temperature roasting/frying, and mid- or high-temperature pyrolysis). While there is no clear cut point of temperature settings for heat processing, thermogravic observations would be helpful in understanding the chemicophysical changes of the cottonseed under a given heating temperature range. Such increased knowledge derived from this work would help in optimizing the heating processes (e.g., roasting and frying) of cottonseed kernels for enhanced food applications and pyrolysis strategies of unconsumable cottonseed or defatted meals for bio-oil and biochar production.

## Figures and Tables

**Table 1:** Selected chemical components of Gd and Gl cottonseed kernels. Data are reported on a dry weight basis with average (A) and standard deviation (SD, n = 3). ADF, acid detergent fiber. ADL, acid detergent lignin.

(A) Major component (g kg^−1^)							
	Moisture	Gossypol *** ^1^	Oil	Protein **	ADF	ADL	Starch **
Gd	67.9 ± 0.5	3.75 ± 0.02	387 ± 18	397 ± 8	100 ± 18	52.3 ± 10.1	12.2 ± 1.0
Gl	68.3 ± 0.2	0.06 ± 0.00	350 ± 14	421 ± 6	109 ± 23	67.8 ± 15.6	16.6 ± 0.4
(B) Macro element and ash (g kg^−1^)							
	P *	Ca	K	Mg	Na	S	Ash
Gd	9.8 ± 0.8	2.0 ± 0.3	12.0 ± 0.7	5.4 ± 0.4	0.6 ± 0.0	4.5 ± 0.3	46.7 ± 0.8
Gl	11.5 ± 0.6	2.3 ± 0.2	12.8 ± 0.5	6.1 ± 0.3	0.6 ± 0.0	4.9 ± 0.2	47.9 ± 0.7
(C) Trace element (mg kg^−1^)							
	Fe	Zn	Cu	Mn	B	Ni	Al
Gd	104 ± 43	70.5 ± 8.2	18.1 ± 1.1	12.9 ± 1.2	13.8 ± 1.1	1.9 ± 0.3	91.6 ± 61.2
Gl	111 ± 3	74.3 ± 2.5	19.0 ± 1.2	13.3 ± 0.7	14.2 ± 0.5	2.1 ± 0.1	109.8 ± 8.4

^1^ Symbols *, **, and *** indicate the values between Gd and Gl samples significantly differed at *p* ≤ 0.05, 0.01 and 0.001, respectively.

**Table 2:** Thermogravimetric and differential thermogravimetric data for Gd and Gl cottonseed kernels. *T*: temperature; *WL*: weight loss; *_o_*: onset decomposition; *_m_*: maximum decomposition rate; *_e_*: end decomposition. Data are presented as the average of triplicate measurements with standard deviation in parentheses. Symbol *, and ns at the data in row Gl-k and Gl-r indicate the values of the Gl samples significantly and not significantly different (*p* = 0.05), respectively, from their corresponding Gd-k and Gd-r samples.

	T_o_ (°C)	WL_o_ (%)	T_m_ (°C)	WL_m_ (%)	T_e_ (°C)	WL_e_ (%)	Char (%) ^1^
Gd-k	143.5 (1.2)	5.9 (0.7)	368.4 (0.8)	55.9 (0.5)	471.7 (1.2)	80.3 (0.7)	16.1 (1.5)
Gl-k	147.1 * (1.3)	5.5 ns (0.9)	368.4 ns (0.7)	52.9 * (0.7)	468.7 * (1.5)	82.2 * (1.1)	15.6 ns (1.1)
Gd-r	177.9 (1.2)	7.6 (1.0)	313.2 (0.9)	36.8 (1.1)	504.9 (1.2)	72.9 (1.2)	25.3 (1.2)
Gl-r	183.6 * (1.0)	9.1 ns (0.8)	314.5 ns (0.9)	37.4 ns (1.0)	504.9 ns (1.0)	72.4 ns (0.9)	25.7 ns (1.3)

^1^, char yield measured at 600 °C.

## Data Availability

The data presented in this study are available upon request from the corresponding author.

## Sample Availability

Samples of the cottonseed kernels with limited quantity are available from the authors.
